# Quality assurance of surgery in the randomized ST03 trial of perioperative chemotherapy in carcinoma of the stomach and gastro‐oesophageal junction

**DOI:** 10.1002/bjs.11184

**Published:** 2019-07-03

**Authors:** W. H. Allum, E. C. Smyth, J. M. Blazeby, H. I. Grabsch, S. M. Griffin, S. Rowley, F. H. Cafferty, R. E. Langley, D. Cunningham

**Affiliations:** ^1^ Gastrointestinal Unit Royal Marsden NHS Foundation Trust London UK; ^2^ Medical Research Council Clinical Trials Unit at University College London London UK; ^3^ Bristol Centre for Surgical Research Bristol Medical School, University of Bristol Bristol UK; ^4^ Department of Gastrointestinal Surgery Royal Victoria Infirmary Newcastle upon Tyne UK; ^5^ Pathology and Data Analytics, Leeds Institute of Medical Research at St James's, School of Medicine University of Leeds Leeds UK; ^6^ Department of Pathology, GROW School for Oncology and Developmental Biology Maastricht University Medical Centre Maastricht The Netherlands

## Abstract

**Background:**

The UK Medical Research Council ST03 trial compared perioperative epirubicin, cisplatin and capecitabine (ECX) chemotherapy with or without bevacizumab (B) in gastric and oesophagogastric junctional cancer. No difference in survival was noted between the arms of the trial. The present study reviewed the standards and performance of surgery in the context of the protocol‐specified surgical criteria.

**Methods:**

Surgical and pathological clinical report forms were reviewed to determine adherence to the surgical protocols, perioperative morbidity and mortality, and final histopathological stage for all patients treated in the study.

**Results:**

Of 1063 patients randomized, 895 (84·2 per cent) underwent resection; surgical details were available for 880 (98·3 per cent). Postoperative assessment data were available for 873 patients; complications occurred in 458 (52·5 per cent) overall, of whom 71 (8·1 per cent) developed complications deemed to be life‐threatening by the responsible clinician. The most common complications were respiratory (211 patients, 24·2 per cent). The anastomotic leak rate was 118 of 873 (13·5 per cent) overall; among those who underwent oesophagogastrectomy, the rate was higher in the group receiving ECX‐B (23·6 per cent *versus* 9·9 per cent in the ECX group). Pathological assessment data were available for 845 patients. At least 15 nodes were removed in 82·5 per cent of resections and the median lymph node harvest was 24 (i.q.r. 17–34). Twenty‐five or more nodes were removed in 49·0 per cent of patients. Histopathologically, the R1 rate was 24·9 per cent (208 of 834 patients). An R1 resection was more common for proximal tumours.

**Conclusion:**

In the ST03 trial, the performance of surgery met the protocol‐stipulated criteria. Registration number: NCT00450203 ( http://www.clinicaltrials.gov).

## Introduction

Surgeons in East Asia established the extent of resection of the stomach and the philosophy of extended lymphadenectomy^1^. However, whether this converts to a survival benefit in all patients has been controversial. Surgical resection for gastric and oesophagogastric junctional (OGJ) cancer in non‐Asian countries has also been variable in extent. Most European studies have demonstrated limited benefit for extended lymphadenectomy^2^, ^3^, with the exception of data from the long‐term follow‐up in the Dutch trial^4^ which confirmed fewer cancer deaths in the group treated by D2 dissection. Surgery for OGJ cancers has evolved based on the classification described by Siewert and colleagues^5^, in which patterns of lymph node spread were defined according to tumour origin in relation to the OGJ. Although some have promoted abdominal, mediastinal and cervical nodal dissection, particularly for squamous cell carcinoma^6^, most surgeons combine abdominal and posterior mediastinal nodal dissection into a so‐called two‐field lymphadenectomy^7^.

This lack of consistency has led to global variability in surgical approach and individual surgeon practice. National guidelines^8^, ^9^, based on evidence and expert opinion, have been designed to promote consistency of surgical practice and have recommended D2 dissection and two‐field lymphadenectomy in appropriately selected patients with gastric and OGJ cancer respectively. Another approach to assess and implement consistency of surgery is to conduct multicentre trials where the type and extent of surgery is prespecified in the trial protocol. In the MAGIC (Medical Research Council (MRC) Adjuvant Gastric Infusional Chemotherapy) trial^10^, surgeons were recommended to resect local and regional perigastric lymph nodes, and at least sample more distant lymph nodes for staging. Based on this recommendation, the D2 resection rate in MAGIC was 41 per cent.

The MRC ST03 trial^11^ was designed to evaluate the addition of bevacizumab, which targets vascular endothelial growth factor, to the combination chemotherapy regimen used in the MAGIC trial. The primary outcome was overall survival, with secondary outcomes of macroscopic disease‐free survival, progression‐free survival, response rates to preoperative chemotherapy and curative (R0) resection rates. The study protocol described recommended surgical procedures and stipulated resection of a minimum of 15 lymph nodes from specified lymph node stations in order to meet the pathological staging requirements of the UICC TNM classification seventh edition^12^, with further nodal dissection according to the individual surgeon's discretion.

The aim of this report was to examine compliance with the surgical protocol in the ST03 trial, and to review whether surgical procedure influenced outcome, irrespective of perioperative treatment group. This is particularly important in view of the overall similar outcomes between the trial arms, as differences in surgical procedures could have confounded any treatment effect in the trial.

## Methods

The MRC ST03 trial was a randomized phase II–III open‐label comparison of perioperative epirubicin, cisplatin and capecitabine (ECX) with ECX and bevacizumab (ECX‐B) in patients with operable oesophagogastric adenocarcinoma. The trial began in 2007, completed recruitment in 2014 and recruited 1063 patients; no significant difference in overall survival was demonstrated between the two arms of the trial^11^.

### Surgery

At trial entry, all patients were considered operable and fit for surgical resection. Eligibility criteria have been detailed previously^11^. Patients entered into the trial included those with adenocarcinoma of the stomach, OGJ (Siewert types I, II and III) and lower oesophagus. Staging at diagnosis required spiral or multislice CT with laparoscopy for all patients with gastric tumours and Siewert II and III OGJ cancers, as well as for patients with Siewert type I and lower oesophageal cancers if indicated clinically. Endoscopic ultrasound examination was recommended for all lower oesophageal and Siewert type I, II or III cancers, and performed according to local practice for all other cancers. PET–CT and MRI were recommended where clinically indicated according to local practice. Eligible stages were based on the sixth edition of the TNM classification^13^ (stomach, Siewert type III OGJ: stage Ib, II, III and IV with no evidence of distant metastases; oesophagus (lower third, Siewert types II and III): stage II to IVa), including those with positive coeliac axis nodes if the surgeon believed that R0 resection could be achieved. The trial protocol stipulated pathological staging according to the TNM sixth edition initially, but this was changed to the seventh edition^12^ during the trial with appropriate protocol amendment. For patients treated with ECX, surgery was planned for 5–6 weeks after the completion of preoperative chemotherapy; for those who had ECX‐B, surgery was scheduled 8 weeks after the last dose of bevacizumab.

### Recommended surgical procedures

At the time of trial initiation, oesophageal and gastric cancer surgery had been centralized in specialist centres across the UK, with experienced surgeons working in multidisciplinary teams that specialized in the management of patients with upper gastrointestinal cancer. Many surgeons and oncologists had taken part in the previous ST01^2^, ST02 (MAGIC)^10^ and OE02^14^ trials. In view of this experience, the protocol did not mandate any further quality assessment other than the following procedure descriptions^15^.

For gastric and Siewert type III cancers, acceptable resection methods included proximal gastrectomy for Siewert type III cancers and cancers of the cardia; total gastrectomy for Siewert type III cancers and cancers of the cardia, fundus or body; and distal subtotal gastrectomy for cancers of the antrum. Combined resection of other organs was permitted if required to achieve complete macroscopic tumour resection. In both total and distal gastrectomy, the greater omentum was removed. For Siewert type II cancers, either extended gastrectomy or two‐phase oesophagogastrectomy was recommended at the surgeon's discretion. For lower oesophageal and Siewert type I OGJ tumours, oesophagogastrectomy, either as a two‐phase right thoracoabdominal approach or a left thoracoabdominal approach, was recommended. Removal of sufficient crural fibres and a cuff of diaphragm, together with the pericardial fat pad and adjacent strips of parietal pleura, was recommended to minimize the risk of a positive radial resection margin.

Acceptable methods of reconstruction according to the surgeon's preference included oesophagogastrostomy, oesophagojejunostomy (Roux‐en‐Y), oesophagojejunogastrostomy (jejunal interposition), gastrojejunostomy (Roux‐en‐Y or Billroth II) and gastrojejunoduodenostomy (jejunal interposition). Anastomotic techniques included handsewn, stapled or combined approaches.

The recommended extent of lymphadenectomy was determined by the location of the primary tumour and the type of resection. The protocol mandated the removal of additional lymph nodes from other lymph node stations to ensure that the total number of lymph nodes excised exceeded 15. A formal D2 dissection was the preferred option for gastric and Siewert type III cancers, although D3–4 dissections were allowed (*Table* S1, supporting information). A two‐field lymphadenectomy was described in the protocol for lower oesophageal and Siewert type I and II cancers. This included removal of lymph node stations 1, 2, 3, 7, 8 and 11 via the abdomen. In the thoracic phase, paraoesophageal and diaphragmatic lymph nodes (stations 108, 110 and 111) should ideally be removed *en bloc* in continuity with the lower oesophagus and lymph nodes at the tracheal bifurcation and along the right and left main bronchi to the pulmonary hilus (stations 107 and 109). The surgical clinical report form required recording of the extent of lymph node dissection as one group of lymph node stations and did not allow for dissection of multiple stations (*Table* S2, supporting information).

The option of a minimally invasive approach was included if specific eligibility criteria for this type of procedure were met. The Trial Management Group (TMG) determined that, for totally minimally invasive procedures, surgeons should provide summary evidence of their previous 20 minimally invasive operations, including total gastrectomy and oesophagogastrectomy, with details of lymph node yields in both the abdomen and the mediastinum and postoperative complication rates. A hybrid approach of an open chest procedure combined with a laparoscopic abdominal procedure was permitted for lower oesophageal, and Siewert type I and II OGJ cancers, without review of previous cases.

### Pathology

The ST03 trial protocol included a detailed briefing document for local pathologists to ensure standardized local pathology procedures and high‐quality pathology in all centres. Pathological staging was initially done according to the sixth edition of the TNM classification^13^, but changed to the seventh edition^12^ once this had been published, with appropriate trial protocol modification. Pathological reporting of pretreatment biopsy and resection specimens was based largely on the WHO classification of digestive tumours (4th edition)^16^, the Royal College of Pathologists data sets for oesophageal and gastric cancer^17^, ^18^ and other relevant literature. As a result, the trial pathology reporting pro forma required details of T category (including macroscopic and histological detail), N status (based on number of nodes examined and number positive) and histologically confirmed distant metastases. In addition, assessment of tumour response using the Mandard system^19^ and resection margin involvement (longitudinal and circumferential, defined as viable tumour within 1 mm of the margin) was required. Following full reporting of the pathology of the resected specimen and review at the local multidisciplinary team meeting, centres were asked to send all haematoxylin and eosin‐stained histological slides of pretreatment biopsies and the resected specimens, together with the respective reports and photographic documentation, for central pathological review.

### Analysis

Details of surgery and the postoperative clinical course were recorded prospectively on case report forms, which were analysed at the MRC Clinical Trials Unit at University College London.

All toxicity in the trial was reported according to Common Terminology Criteria for Adverse Events (CTC‐AE) version 3.0^20^, then grouped by organ system. Specific surgery‐related postoperative complications in the data set included intraoperative events, anastomotic leak (clinical *versus* radiological), return to surgery, intestinal obstruction, pancreatic fistula and intra‐abdominal sepsis. Anastomotic leaks were recorded as clinical, radiological/endoscopic or both clinical and radiological/endoscopic. General postoperative complications included cardiorespiratory and thromboembolic events, sepsis and renal dysfunction. Pathology‐related outcomes were measured using lymph node yield and resection margin involvement.

All data were tabulated and summarized (with either percentages or median (i.q.r.), as appropriate) according to treatment group and in the trial as a whole. No comparative statistical tests were undertaken as the trial was not powered for these outcomes, and this was not the purpose of the present analysis.

## Results

**Figure 1:**
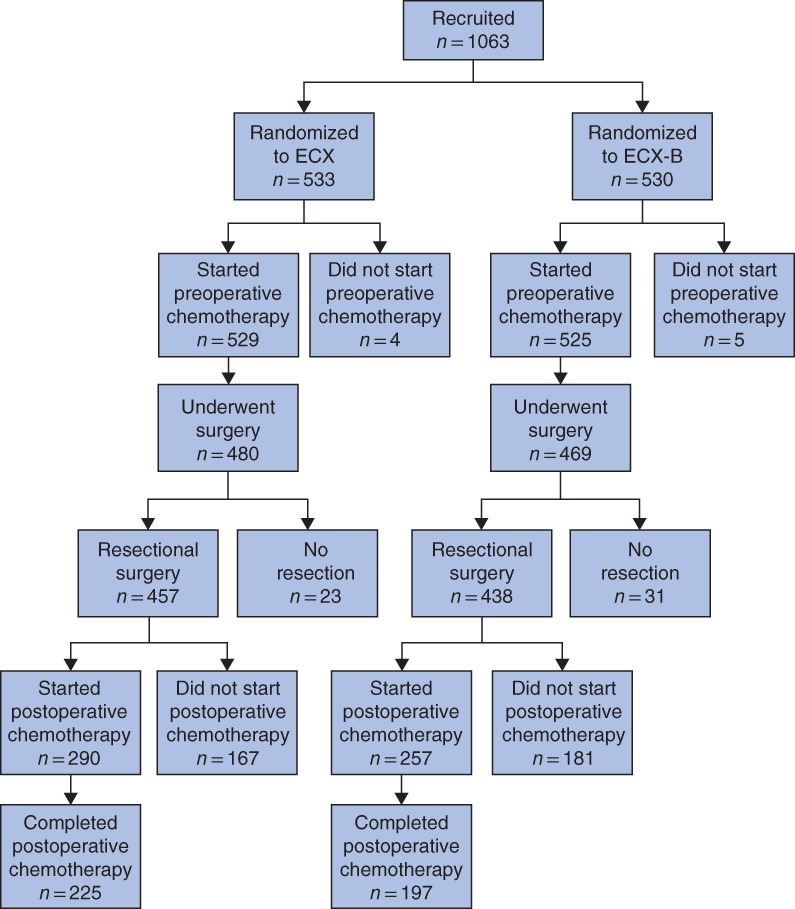
Flow diagram for ST03 trialECX, epirubicin, cisplatin and capecitabine; B, bevacizumab.

Of 895 patients who underwent resectional surgery in the ST03 trial (84·2 per cent of 1063 randomized), surgical details were available for 880 (98·3 per cent) (*Fig*. 1 and *Table* 1). There was no difference in surgical approaches across the arms of the trial. Open surgery was performed in 547 patients (62·2 per cent), and a minimally invasive approach in 222 (25·2 per cent), which was either a totally minimally invasive procedure (49, 5·6 per cent), a hybrid approach (laparoscopic abdomen and open thoracotomy: 110, 12·5 per cent) or a laparoscopically assisted procedure (63, 7·2 per cent). Data on surgical approach were missing for 77 patients (8·8 per cent). Extended lymphadenectomy including perigastric, left gastric, hepatic and splenic artery lymph nodes was performed according to the surgeon's discretion in 46·6 per cent of patients, which was similar in both trial arms (*Table* S2, supporting information).

**Table 1:** Surgical procedures and techniques

	ECX (*n* = 450)	ECX‐B (*n* = 430)	Total (*n* = 880)
**Surgical procedure**			
Oesophagogastrectomy	235 (52·2)	224 (52·1)	459 (52·2)
Total gastrectomy	142 (31·6)	130 (30·2)	272 (30·9)
Subtotal gastrectomy	16 (3·6)	17 (4·0)	33 (3·8)
Proximal gastrectomy	1 (0·2)	1 (0·2)	2 (0·2)
Distal gastrectomy	44 (9·8)	44 (10·2)	88 (10·0)
Other	12 (2·7)	14 (3·3)	26 (3·0)
**Surgical technique**			
Open surgery	275 (61·1)	272 (63·3)	547 (62·2)
Laparoscopically assisted surgery	36 (8·0)	27 (6·3)	63 (7·2)
Laparoscopic abdomen and open chest	59 (13·1)	51 (11·9)	110 (12·5)
Laparoscopic abdomen and thoracoscopic chest	26 (5·8)	23 (5·3)	49 (5·6)
Other	12 (2·7)	22 (5·1)	34 (3·9)
Missing	42 (9·3)	35 (8·1)	77 (8·8)

Values in parentheses are percentages. ECX, epirubicin, cisplatin and capecitabine; B, bevacizumab.

Sites undertaking total minimally invasive laparoscopic operations routinely within the trial were required to provide information on their previous experience with these techniques. Evidence was submitted for 67 of 112 operations at 14 sites. Of the remaining 45 operations, five were performed just once by centres and noted as a protocol deviation. The surgical requirements were followed by sites in 95·5 per cent of resection procedures.

Some 159 patients completed preoperative chemotherapy but did not undergo resection. Fifty‐four of these patients had surgery, which comprised a laparotomy only (51 patients) or a laparotomy and bypass procedure (3 patients). The remaining patients did not undergo surgery because of disease progression, patient preference or for a number of other reasons.

### Postoperative complications

Postoperative morbidity and mortality after resection are summarized in *Table* 2. Postoperative assessment details were available for 97·5 per cent of operated patients (873 of 895). There were 24 deaths (2·7 per cent) within 30 days of surgery and 33 (3·7 per cent) within 90 days. There were no deaths within 30 days among those who underwent laparotomy alone and were found to be inoperable (*Table* S3, supporting information).

**Table 2:** Summary of postoperative complications

	ECX (*n* = 446)	ECX‐B (*n* = 427)	Total (*n* = 873)
**All postoperative complications (maximum severity)**			
None	231 (51·8)	184 (43·1)	415 (47·5)
Non‐life‐threatening	178 (39·9)	209 (48·9)	387 (44·3)
Life‐threatening	37 (8·3)	34 (8·0)	71 (8·1)
**Notable events (maximum severity)**			
None	369 (82·7)	330 (77·3)	699 (80·1)
Non‐life‐threatening	62 (13·9)	81 (19·0)	143 (16·4)
Life‐threatening	15 (3·4)	16 (3·7)	31 (3·6)
**Other complications, not notable events (maximum severity)**			
None	245 (54·9)	213 (49·9)	458 (52·5)
Non‐life‐threatening	172 (38·6)	187 (43·8)	359 (41·1)
Life‐threatening	29 (6·5)	27 (6·3)	56 (6·4)
**Revisional operations**			
No	406 (91·0)	390 (91·3)	796 (91·2)
Yes	39 (8·7)	37 (8·7)	76 (8·7)
Unknown	1 (0·2)	0 (0)	1 (0·1)
**Death before discharge from hospital**			
No	432 (96·9)	414 (97·0)	846 (96·9)
Yes	12 (2·7)	11 (2·6)	23 (2·6)
Unknown	2 (0·4)	2 (0·5)	4 (0·5)

Values in parentheses are percentage of patients with postoperative assessment details available; this information was missing for 11 patients who underwent surgery in each group. ECX, epirubicin, cisplatin and capecitabine; B, bevacizumab.

Complications were recorded as wound‐related, including wound healing, wound infection, intra‐abdominal sepsis and haemorrhage, or respiratory, including pleural effusion, chest infection, thromboembolic, cardiac and systemic infection. These were graded simply as life‐threatening or not life‐threatening as judged clinically. There was no apparent difference in the overall rates of complications across the various surgical procedures. Complications of any type occurred in 458 patients (52·5 per cent) (*Table* 3). The most common complications were respiratory (211 patients, 24·2 per cent) and sepsis (158, 18·1 per cent). The most common respiratory complication was respiratory tract infection (143 patients, 16·4 per cent). Wound healing complications were seen in 86 patients (9·9 per cent) and superficial wound infections in 75 (8·6 per cent), and formed the bulk of the infectious complications. Wound healing complications of any grade were more common in patients treated with bevacizumab than in the ECX group: 53 of 427 (12·4 per cent) *versus* 33 of 446 (7·4 per cent) respectively. Rates of respiratory complications were higher among patients undergoing oesophagogastrectomy (145, 32·0 per cent) than for other procedures, whereas rates of septic complications were similarly distributed across patients undergoing oesophagogastrectomy, total or subtotal gastrectomy (*Table* 4). Details about complications for those who had surgery but did not undergo resection were available for 37 of 54 patients (*Table* S3, supporting information). Nine of these patients developed complications, which were cardiorespiratory or wound‐related.

**Table 3:** Postoperative complications by type

	ECX (*n* = 446)	ECX‐B (*n* = 427)	Total (*n* = 873)
**Wound healing complications**			
No	413 (92·6)	374 (87·6)	787 (90·1)
Present but not life‐threatening	30 (6·7)	48 (11·2)	78 (8·9)
Life‐threatening	3 (0·7)	5 (1·2)	8 (0·9)
**Superficial wound infection**			
No	409 (91·7)	389 (91·1)	798 (91·4)
Present but not life‐threatening	35 (7·8)	37 (8·7)	72 (8·2)
Life‐threatening	2 (0·4)	1 (0·2)	3 (0·3)
**Deep wound infection**			
No	431 (96·6)	415 (97·2)	846 (96·9)
Present but not life‐threatening	12 (2·7)	8 (1·9)	20 (2·3)
Life‐threatening	3 (0·7)	4 (0·9)	7 (0·8)
**Intra‐abdominal sepsis**			
No	428 (96·0)	410 (96·0)	838 (96·0)
Present but not life‐threatening	11 (2·5)	10 (2·3)	21 (2·4)
Life‐threatening	7 (1·6)	7 (1·6)	14 (1·6)
**Haemorrhage requiring transfusion or intervention**			
No	433 (97·1)	414 (97·0)	847 (97·0)
Present but not life‐threatening	9 (2·0)	6 (1·4)	15 (1·7)
Life‐threatening	4 (0·9)	7 (1·6)	11 (1·3)
**Pleural effusion requiring treatment**			
No	396 (88·8)	386 (90·4)	782 (89·6)
Present but not life‐threatening	45 (10·1)	35 (8·2)	80 (9·2)
Life‐threatening	5 (1·1)	6 (1·4)	11 (1·3)
**Empyema**			
No	437 (98·0)	409 (95·8)	846 (96·9)
Present but not life‐threatening	8 (1·8)	14 (3·3)	22 (2·5)
Life‐threatening	1 (0·2)	4 (0·9)	5 (0·6)
**Respiratory failure**			
No	419 (93·9)	404 (94·6)	823 (94·3)
Present but not life‐threatening	14 (3·1)	10 (2·3)	24 (2·7)
Life‐threatening	13 (2·9)	13 (3·0)	26 (3·0)
**Respiratory tract infection**			
No	374 (83·9)	356 (83·4)	730 (83·6)
Present but not life‐threatening	66 (14·8)	64 (15·0)	130 (14·9)
Life‐threatening	6 (1·3)	7 (1·6)	13 (1·5)
**Pulmonary embolism**			
No	439 (98·4)	418 (97·9)	857 (98·2)
Present but not life‐threatening	5 (1·1)	8 (1·9)	13 (1·5)
Life‐threatening	2 (0·4)	1 (0·2)	3 (0·3)
**Deep vein thrombosis**			
No	443 (99·3)	420 (98·4)	863 (98·9)
Present but not life‐threatening	3 (0·7)	6 (1·4)	9 (1·0)
Life‐threatening	0 (0)	1 (0·2)	1 (0·1)
**Cardiac complications**			
No	423 (94·8)	397 (93·0)	820 (93·9)
Present but not life‐threatening	15 (3·4)	24 (5·6)	39 (4·5)
Life‐threatening	8 (1·8)	6 (1·4)	14 (1·6)
**MRSA**			
No	437 (98·0)	421 (98·6)	858 (98·3)
Present but not life‐threatening	9 (2·0)	6 (1·4)	15 (1·7)
**Other**			
No	337 (75·6)	309 (72·4)	646 (74·0)
Present but not life‐threatening	95 (21·3)	100 (23·4)	195 (22·3)
Life‐threatening	14 (3·1)	18 (4·2)	32 (3·7)

Values in parentheses are percentage of patients with postoperative assessment details available; this information was missing for 11 patients who underwent surgery in each group. ECX, epirubicin, cisplatin and capecitabine; B, bevacizumab; MRSA, methicillin‐resistant *Staphylococcus aureus*.

**Table 4:** Postoperative complications by procedure

	Oesophagogastrectomy (*n* = 453)	Total gastrectomy (*n* = 272)	Subtotal gastrectomy* (*n* = 123)	Other (*n* = 25)
Sepsis	80 (17·7)	51 (18·8)	20 (16·3)	7 (28)
Respiratory	145 (32·0)	45 (16·5)	12 (9·8)	9 (36)
Bleeding†	17 (3·8)	6 (2·2)	2 (1·6)	1 (4)
Thromboembolic	15 (3·3)	4 (1·5)	5 (4·1)	1 (4)
Cardiovascular	38 (8·4)	9 (3·3)	4 (3·3)	2 (8)
Other	137 (30·2)	57 (21·0)	21 (17·1)	12 (48)

Values in parentheses are percentages.

* Includes subtotal, distal and proximal gastrectomies.

† Requiring transfusion or intervention.

### Surgical complications

Two specific indicators of technical complications were evaluated: anastomotic leaks and return to theatre. Combining both trial arms, the anastomotic leak rate was 13·5 per cent (118 of 873 patients with postoperative data available) (*Table* 5). Twenty‐six patients had clinical leaks, 25 leaks were identified radiologically or endoscopically, and 65 were diagnosed clinically and confirmed radiologically or endoscopically; in two patients the evidence for a leak was unclear. After oesophagogastrectomy, the anastomotic leak rate was higher in those randomized to ECX‐B than in the ECX group: 52 of 220 (23·6 per cent) *versus* 23 of 233 (9·9 per cent) respectively. There was no difference between groups in leak rates after any type of gastrectomy. The anastomotic leak rate was 10·2 per cent (43 of 420) in patients undergoing a procedure other than oesophagogastrectomy. The majority of leaks (80 of 118, 67·8 per cent) occurred within the first 10 days after surgery.

**Table 5:** Anastomotic leak rates

	ECX (*n* = 446)	ECX‐B (*n* = 427)	Total (*n* = 873)
**By tumour site at randomization**			
Lower oesophageal	7 of 65 (11)	16 of 59 (27)	23 of 124 (18·5)
OGJ, type I	5 of 57 (9)	10 of 53 (19)	15 of 110 (13·6)
OGJ, type II	11 of 87 (13)	12 of 71 (17)	23 of 158 (14·6)
OGJ, type III	6 of 75 (8)	19 of 88 (22)	25 of 163 (15·3)
Stomach	14 of 162 (8·6)	18 of 156 (11·5)	32 of 318 (10·1)
**By surgical procedure**			
Oesophagogastrectomy	23 of 233 (9·9)	52 of 220 (23·6)	75 of 453 (16·6)
Total gastrectomy	18 of 139 (13·0)	19 of 129 (14·7)	37 of 268 (13·8)
Subtotal gastrectomy	0 of 16 (0)	1 of 17 (6)	1 of 33 (3)
Distal gastrectomy	1 of 43 (2)	2 of 43 (5)	3 of 86 (3)
Other	1 of 15 (7)	1 of 18 (6)	2 of 33 (6)
**By surgical procedure (combined)**			
Oesophagogastrectomy	23 of 233 (9·9)	52 of 220 (23·6)	75 of 453 (16·6)
All other procedures	20 of 213 (9·4)	23 of 207 (11·1)	43 of 420 (10·2)
			
**Overall**	43 of 446 (9·6)	75 of 427 (17·6)	118 of 873 (13·5)

Values in parentheses are percentages. ECX, epirubicin, cisplatin and capecitabine; B, bevacizumab; OGJ, oesophagogastric junctional.

Seventy‐six patients (8·7 per cent) had to return to the operating theatre in the initial postoperative period; this occurred in the first week in 38 patients (50 per cent) and in the second week in a further 20 (26 per cent). The commonest reasons for reoperation were anastomotic leak (18 patients, 24 per cent), and laparotomy (17, 22 per cent) or thoracotomy (12, 16 per cent) for lavage to treat sepsis.

### Surgical pathology

Details of pathological staging and response to chemotherapy have been described previously^11^. Pathology results were available for 845 patients (94·4 per cent of those who underwent tumour resection). The median number of lymph nodes dissected from the resection specimen by the local pathologist was 24 (i.q.r. 17–34); this was similar in both arms of the trial. At least 15 lymph nodes were retrieved in 82·5 per cent of resection specimens, and 25 or more lymph nodes in 49·0 per cent of specimens (*Table* 6).

**Table 6:** Total number of lymph nodes retrieved from the resected specimen according to local pathologists

Total no. of lymph nodes	ECX (*n* = 436)	ECX + B (*n* = 409)	Total (*n* = 845)
Median (i.q.r.)	24 (17–33)	25 (18–34)	24 (17–34)
Range	0–96	0–89	0–96
0	5 (1·1)	5 (1·2)	10 (1·2)
< 15	74 (17·0)	57 (13·9)	131 (15·5)
15–24	146 (33·5)	137 (33·5)	283 (33·5)
25–34	109 (25·0)	110 (26·9)	219 (25·9)
35–44	56 (12·8)	60 (14·7)	116 (13·7)
≥ 45	42 (9·6)	37 (9·0)	79 (9·3)
Unknown	4 (0·9)	3 (0·7)	7 (0·8)

Values in parentheses are percentages unless indicated otherwise. ECX, epirubicin, cisplatin and capecitabine; B, bevacizumab.

Information on pathological resection margin status was available for 834 patients; 626 of these patients (75·1 per cent) had an R0 resection and 208 (24·9 per cent) had a pathologically positive resection margin (R1) (*Table* S4, supporting information). The majority with an R1 margin (146 patients) had an oesophagogastrectomy, and 132 of these patients had a positive circumferential (radial) margin. This equated to an R1 resection rate of 31·8 per cent for all of the oesophagogastric resections. The remaining R1 resections included proximal resection margin involvement in 42 patients (20·2 per cent of all R1 resections) and distal margin involvement in 33 (15·9 per cent).

## Discussion

The ST03 trial was a national multicentre trial that recruited patients at almost 100 hospitals in the UK between 2007 and 2013. Its primary outcome was overall survival between the two treatment arms. The trial had a pragmatic design, with R0 resection as the only secondary outcome measure with a surgical theme. As a result, specific details of surgical procedures were not recorded as in a trial comparing surgical procedures. This trial therefore delivers a snapshot of UK contemporary surgical practice. The ST03 surgical protocol was designed to prespecify the surgical approaches and extent of resection to minimize performance bias related to individual surgeon preference, which may be influenced by tumour response to perioperative chemotherapy. The study included prospective data collection on case report forms to record the type of surgical procedure actually undertaken. Development of the ST03 trial protocol was informed by best current evidence. However, it was also pragmatic, thus allowing flexibility in several components of the intervention (such as minimal access or open surgery, extent of lymph node dissection). High‐quality pathological data were available to relate surgical procedures to outcome measures, such as lymph node yield and resection margin status, owing to use of a prespecified histopathology protocol, which included detailed guidance for local pathologists as well as a comprehensive prospective data collection. This analysis showed that surgical procedures and outcomes were similar in both arms of the trial. Therefore, heterogeneity of surgical approach was unlikely to be responsible for the lack of benefit observed in the experimental arm of the trial.

The inclusion of minimally invasive surgery required a protocol modification as such techniques were not commonplace at the inception of the trial. The TMG required surgeons undertaking these procedures in patients entered into the trial to provide evidence of their qualitative outcomes. Despite this requirement, formal review by the TMG was completed for only 28·1 per cent of the minimally invasive procedures undertaken. Retrospective review of the remaining patients showed no excess complication rates and equivalent lymph node yields, suggesting consistency of the quality of the surgery. However, this does highlight one of the challenges in a randomized trial where protocol modifications may be required that could affect outcome.

Despite comprehensive staging assessments at the time of diagnosis and randomization into the trial, 15·8 per cent of the trial population did not undergo resection, with 5·1 per cent found to be inoperable at laparotomy. The outcome for this group was not complicated by excess postoperative morbidity. These levels of inoperability and unresectability are important findings, which should be explained to patients when entering into a trial^21^.

Surgeons performing operations and pathologists dissecting resection specimens within the context of the ST03 trial were highly compliant with respect to the protocol requirement of resecting and retrieving a minimum of 15 lymph nodes; this goal was achieved in 82·5 per cent of resections. Furthermore 25 or more lymph nodes were retrieved by the pathologist in 49·0 per cent of resections. Although pathological examination of the resected specimens in the trial was based on Royal College of Pathologists guidance, there are limitations in this data as lymph node stations were not examined individually in the majority of patients. Nevertheless, these lymph node harvest figures show a definite increase compared with those in the MAGIC trial^10^, in which 53 per cent of patients had more than 15 nodes removed and 19 per cent had more than 25 excised. Notably, in the MAGIC trial, surgeons undertook procedures at their discretion; the extent of lymphadenectomy was not specified in the MAGIC trial protocol. Moreover, these lymph node yield figures are higher than those reported in the Dutch CRITICS^22^ trial, in which 72·8 per cent of resections were compliant with the protocol stipulation of sampling a minimum of 15 nodes and 87·5 per cent of patients had at least a D1+ resection. Furthermore, the median number of nodes examined in CRITICS was 20 (range 0–72) contrasting with 24 (0–96) in ST03.

Surgery in the ST03 study was associated with a low rate of postoperative mortality which, at 3·7 per cent at 90 days, is a further improvement in comparison with both the MAGIC trial (ST02), in which the 90‐day mortality rate was 6 per cent, and the MRC ST01 study, with hospital mortality rates of 9 and 16 per cent after D1 and D2 total gastrectomy respectively. The postoperative mortality rate in ST03 was similar to that reported for oesophagectomy and gastrectomy in the UK National Oesophago‐Gastric Cancer Audit (NOGCA)^23^, which records approximately 98 per cent of cases resected annually in the UK.

Despite these improvements in postoperative mortality compared with earlier trials, there was a relatively high rate of postoperative complications in ST03 (52·5 per cent of all patients). However, this figure describes all levels of the spectrum of complications, including many that did not require clinical intervention. This represents a limitation of the present study and is a reflection of the protocol, which was developed before more specific definition of complications was standardized. The authors therefore advocate use of a systematic classification such as the Clavien–Dindo classification^24^ in future trials as well as consensus guidance on recording complications^25^, ^26^. In addition, patients undergoing oesophageal and gastric cancer resection have established co‐morbidity, and optimization of pulmonary function should be standard practice in the context of prehabilitation and enhanced recovery approaches to minimize predictable postoperative complications.

Overall compliance with the protocol in ST03 represents a definite improvement in surgical and pathology practice compared with previous studies. However, it is important to recognize that surgery within the ST03 trial was subject to a number of other significant changes in practice. The trial was undertaken during the time of reconfiguration of surgical services for oesophageal and gastric cancer in the UK, which included greater prominence of multidisciplinary team management for all patients. In addition, there was enhanced surgical and pathology specialization as well as advances in critical and intensive care treatments. The inclusion of minimally invasive techniques required a careful review of practice and outcomes for those wishing to use these procedures in the context of their established expertise. The majority of surgeons had contributed to previous multicentre studies (ST01, ST02 and OE05). It was acknowledged that their adherence to the protocol would not be problematic, and specific evidence of their practice and outcomes was not considered necessary. Data from audits have clearly shown improvements and consistency in overall outcome with the emphasis on quality assurance and publication of individual‐surgeon outcomes. The pragmatic nature of this trial has shown how large multicentre trials can be undertaken effectively with consistent outcomes.

Two challenges are highlighted by the ST03 results. The first of these, the high rate of anastomotic leak in patients who underwent oesophagogastrectomy after treatment with bevacizumab (23·6 per cent), is unique to the present trial. This inflated the overall rate of anastomotic leakage to 13·5 per cent, which compares unfavourably with 7 per cent in the NOGCA data set. However, the overall reoperation rate of 8·7 per cent is comparable to rates of 9·8 per cent for oesophagectomy and 8·1 per cent for gastrectomy recorded in the NOGCA^23^. Despite careful evaluation of all potential variables, it was not possible to explain the difference in leak rate between patients who received bevacizumab and those who did not, and it is currently considered to represent a potential adverse effect on local healing secondary to microvascular insufficiency related to treatment with bevacizumab. Consideration of other data and sensitivity analyses did not provide any indication that this excess rate of anastomotic leakage had a substantial effect on the overall primary outcome of the trial; the Independent Data Monitoring Committee had reviewed the data when this rate was identified and stopped recruitment of patients scheduled for oesophagogastrectomy.

The second challenge is the relatively high rate of R1 resections (25·1 per cent) in the trial (gastrectomy 15·7 per cent and oesophagogastrectomy 31·8 per cent). The majority of positive margins (90·4 per cent for oesophagogastrectomies) were circumferential. Similar results have been reported in the contemporaneous OE05 study^27^, in which the R1 rate was 34 per cent. The OE05 protocol specified the extent of dissection at the diaphragmatic hiatus to include resection of a cuff of diaphragm and both parietal pleura adjacent to the OGJ. Compliance with this aspect of the protocol has not been determined in the present trial. However, lower rates of circumferential resection margins have been described in surgical series with more radical procedures. In CROSS (ChemoRadiotherapy for Oesophageal cancer followed by Surgery Study)^28^, the R1 rate in the chemoradiotherapy arm was 8 per cent compared with 30 per cent in the control surgery‐only arm. There is thus a challenge to determine whether more standardized surgery can achieve R0 rates similar to those achieved after neoadjuvant chemoradiotherapy. However, the high R0 rate in the treatment arm of the CROSS trial may reflect the effect of chemoradiotherapy as well as the fact that patients with adenocarcinoma and those with squamous cell carcinoma were included in the trial. Furthermore, more contemporary taxane‐containing preoperative chemotherapy regimens such as FLOT (5‐fluorouracil, oxaliplatin and docetaxel) seem to be associated with improved R0 resection rates compared with the anthracycline‐based regimens used in ST03 and OE05^29^. This is of particular relevance, as the survival benefit of chemoradiotherapy over perioperative chemotherapy is still unclear. Ongoing clinical trials are currently addressing this issue. The results of these studies and assessment of response to neoadjuvant therapy are likely to affect selection of patients who have the greatest chance of benefit from operative intervention.

The present analysis has shown that surgery in the ST03 trial was performed to a higher standard than in previous similar UK trials, in terms of postoperative mortality and lymphadenectomy, which also reflects a higher standard of pathology reporting. This was consistent across both treatment arms, indicating that surgery had no effect on the overall outcome of the trial. The rates of postoperative complications were high, partly reflecting the inclusion of all grades of morbidity, although few patients developed life‐threatening complications. There remains the challenge of achieving a higher rate of R0 resection to complement the benefits of perioperative strategies.

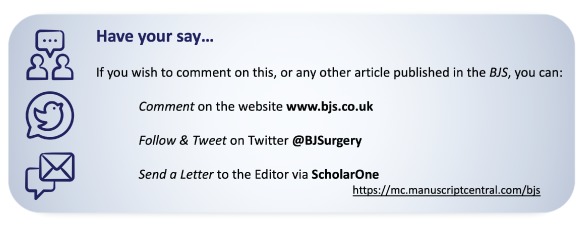

## Supporting information


**Table S1.** Protocol prescribed nodal stations for excision according to site of primary tumour
**Table S2.** Extent of Lymphadenectomy as reported by surgeon
**Table S3.** Complications after non‐resectional surgery (n = 37 of 54 non‐resected cases) (Some patients had more than one complication)
**Table S4.** R1 Resection
