# Supplementary material for: Quality assurance of surgery in the randomized ST03 trial of perioperative chemotherapy in carcinoma of the stomach and gastro‐oesophageal junction
Source: Br J Surg. 2019 Jul 3;106(9):1204–15. doi: 10.1002/bjs.11184 (PMC6771829; doi:10.1002/bjs.11184)
Supplement: Supplementary file 1 — Table S1. Protocol prescribed nodal stations for excision according to site of primary tumour Table S2. Extent of Lymphadenectomy as reported by surgeon Table S3. Complications after non‐resectional surgery (n = 37 of 54 non‐resected cases) (Some patients had more than one complication) Table S4. R1 Resection [file BJS-106-1204-s001.docx]

**BJS11184**

**Quality assurance of surgery in the randomized ST03 trial of perioperative chemotherapy in carcinoma of the stomach and gastro-oesophageal junction**

W. H. Allum, E. C. Smyth, J. M. Blazeby, H. I. Grabsch, S. M. Griffin, S. Rowley, F. H. Cafferty, R. E. Langley and D. Cunningham

**Table S1** Protocol prescribed nodal stations for excision according to site of primary tumour

|  | Nodal Stations |
| --- | --- |
| Proximal Partial Gastrectomy | 1,2,3,4a, 7,8,11 |
| Distal subtotal gastrectomy | 1,3,4b,5,6,7,8 |
| Total Gastrectomy | 1,2,3,4,5,6,7,8, |
| Oesophago-gastrectomy | 1,2,3,7,8,11,107, 108, 109, 110, 111 |

**Table S2 Extent of Lymphadenectomy as reported by surgeon**

|  | |  | **ECX** | **ECX+B** | **Total** |
| --- | --- | --- | --- | --- | --- |
|  | |  | **n %** | **n %** | **n %** |
|  | |  |  |  |  |
| **Extent of Lymphaenectony** | | None | 12 3 | 17 4 | 29 3 |
|  | | Perigastric nodes only | 52 12 | 58 13 | 110 13 |
|  | Perigastric nodes plus sampling of more distant nodes | | 50 11 | 62 14 | 112 13 |
|  | Clearance of perigastric, left gastric, hepatic & splenic artery nodes | | 223 50 | 187 43 | 410 47 |
|  | Mediastinal nodes | | 84 19 | 68 16 | 152 17 |
|  | | Unknown | 29 6 | 38 9 | 67 8 |
|  | |  |  |  |  |
|  | |  |  |  |  |
| **TOTAL** | |  | **450** | **430** | **880** |

**Table S3 Complications after non-resectional surgery (n=37 of 54 non-resected cases) (Some patients had more than one complication)**

| **Post-operative complications in non-resected patients (n=37)** | | | **N (%)** |
| --- | --- | --- | --- |
| 30-day Mortality | |  | 0 (0) |
| Any post-operative complications | |  | 9 (24) |
|  |  | *Non-Life Threatening* | *9* |
|  |  | *Life-threatening* | *0* |
| Delayed wound healing | |  | 3 (8) |
| Wound sepsis | |  | 3 (8) |
| Respiratory infection | |  | 2 (5) |
| Pleural effusion | |  | 1 (3) |
| Acute coronary syndrome | |  | 1 (3) |
| Urinary tract infection | |  | 1 (3) |
| Deep venous thrombosis | |  | 1 (3) |
| Revisional intervention | |  | 2 (5) |
|  | | *Oesophageal stent* | *1* |
|  | *Laparotomy for small bowel obstruction* | | *1* |
| Median length of postoperative hospital stay (days) | | | 7 (IQR 4-12) |

**Table S4 R1 Resection**

|  | |  | | | | | **ECX** | | **ECX+B** | | **Total** | |
| --- | --- | --- | --- | --- | --- | --- | --- | --- | --- | --- | --- | --- |
|  | | | | |  | | **n %** | | **n %** | | **n %** | |
|  | | | | |  | |  | |  | |  | |
| **POSITIVE MARGINS** | | | |  | | |  | |  | |  | |
| **All resections** | | R1 | | | | | 108 25 | | 100 24 | | 208 25 | |
| (multiple locations may be indicated | | | | | | *Proximal* | *24* | | *18* | | *42* | |
| for a given patient) | | *Distal* | | | | | *17* | | *16* | | *33* | |
|  | |  | | | | |  | |  | |  | |
| **Oesophago-gastrectomy only** | | | | R1 | | | 75 | | 71 | | 146 | |
|  | *at or within 1mm of circumferential* | | | | | | *69* | | *63* | | *132* | |
|  |  | | | | | |  | |  | |  | |
| **R0 resection** | | |  | | | | 321 | 74 | 305 | 75 | 626 | 74 |
| **Not reported** | | |  | | | | 7 | 1 | 4 | 1 | 11 | 1 |
| **TOTAL** | |  | | | | | **436** |  | **409** |  | **845** |  |
